# Protein-directed self-assembly of a fullerene crystal

**DOI:** 10.1038/ncomms11429

**Published:** 2016-04-26

**Authors:** Kook-Han Kim, Dong-Kyun Ko, Yong-Tae Kim, Nam Hyeong Kim, Jaydeep Paul, Shao-Qing Zhang, Christopher B. Murray, Rudresh Acharya, William F. DeGrado, Yong Ho Kim, Gevorg Grigoryan

**Affiliations:** 1 SKKU Advanced Institute of Nanotechnology, Sungkyunkwan University, Suwon 16419, Korea; 2 Department of Electrical and Computer Engineering, New Jersey Institute of Technology, Newark, New Jersey 07102, USA; 3 School of Biological Sciences, National Institute of Science Education and Research, Bhubaneswar, Odisha 752050, India; 4 Department of Pharmaceutical Chemistry and Cardiovascular Research Institute, University of California, San Francisco, California 94158, USA; 5 Department of Chemistry, University of Pennsylvania, Philadelphia, Pennsylvania 19104, USA; 6 Department of Chemistry and Materials Science and Engineering, University of Pennsylvania, Philadelphia, Pennsylvania 19104, USA; 7 Center for Neuroscience Imaging Research, Institute for Basic Science (IBS), Suwon 16419, Korea; 8 Department of Computer Science and Department of Biological Sciences, Dartmouth College, Hanover, New Hampshire 03755, USA

## Abstract

Learning to engineer self-assembly would enable the precise organization of molecules by design to create matter with tailored properties. Here we demonstrate that proteins can direct the self-assembly of buckminsterfullerene (C_60_) into ordered superstructures. A previously engineered tetrameric helical bundle binds C_60_ in solution, rendering it water soluble. Two tetramers associate with one C_60_, promoting further organization revealed in a 1.67-Å crystal structure. Fullerene groups occupy periodic lattice sites, sandwiched between two Tyr residues from adjacent tetramers. Strikingly, the assembly exhibits high charge conductance, whereas both the protein-alone crystal and amorphous C_60_ are electrically insulating. The affinity of C_60_ for its crystal-binding site is estimated to be in the nanomolar range, with lattices of known protein crystals geometrically compatible with incorporating the motif. Taken together, these findings suggest a new means of organizing fullerene molecules into a rich variety of lattices to generate new properties by design.

## Abstract (web summary)

Self-assembly enables complex structures to be fabricated from a few relatively simple components, but requires a detailed understanding of how the constituents may interact. Here, the authors report the rational assembly and crystallographic characterization of a fullerene-protein superstructure.

## Graphical abstract

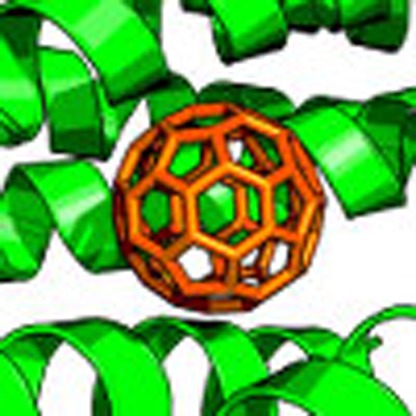

Programmable self-assembly of molecular building blocks is a highly desirable way of achieving bottom-up control over novel functions and materials. Applications of molecular assemblies are well explored in the literature, ranging from optoelectronic^1^,^2^, magnetic^3^, and photovoltaic^4^ devices to chemical and bioanalytical sensing^5^, and medicine^6^. However, it has been a daunting challenge to quantitatively describe and control the driving forces that govern self-assembly, particularly given the broad range of molecular building blocks one would like to organize. In this respect, nature's self-assembling macromolecules hold considerable promise as standard chassis for encoding precise organization. By learning to engineer the assembly of these molecules, myriad other molecular building blocks can be co-organized in desired ways through non-covalent or covalent attachment. The protein polymer is a particularly attractive candidate for a standard assembly chassis given its rich chemical alphabet, diversity of available assembly geometries, broad ability to engage other molecular moieties, and the possibility of engineered function. Considerable progress has been made in the area of engineering protein assemblies^7^,^8^, using either computational^9^,^10^,^11^,^12^,^13^,^14^ or rational approaches^15^,^16^,^17^,^18^, but the problem remains a grand challenge. A major difficulty lies in accounting for the enormous continuum of possible assembly geometries available to proteins to engineer a sequence that predictably prefers just one. General design principles, which provide predictive rules of assembly, are thus of enormous utility in limiting the geometric search space and enabling robust design^11^,^19^.

In this work, we demonstrate the first ever high-resolution structure of co-assembly between a protein and buckminsterfullerene (C_60_), which suggests a simple structural mode for protein–fullerene co-organization. Three separate crystal structures, resolved to 1.67, 1.76 and 2.35 Å, reveal a protein lattice with C_60_ groups occupying periodic sites wedged between two helical segments, each donating a Tyr residue. A half site of the motif is estimated to have nM-scale affinity for C_60_, such that binding of fullerene appears to direct the organization of protein units in the co-crystal. The assembly exhibits a nm-spaced helical arrangement of fullerenes along a crystallographic axis, endowing the crystal with electrical conductance properties. We closely investigate the interfacial geometry of the C_60_-binding motif, finding it to be common among protein crystal lattices. C_60_ and its derivatives have been previously reported to interact with several proteins^20^,^21^,^22^,^23^,^24^,^25^, although a high-resolution structure of a protein–C_60_ has been lacking. Still, prior evidence of interaction indicates that fullerenes and proteins are compatible as materials. This, together with the simple (and naturally recurrent) geometry of the C_60_-binding motif we discover, suggests that it may be possible to use the structural principles emergent from our study to generate a variety of C_60_–protein co-assemblies to further explore and exploit the properties of fullerenes^26^.

## Results

### C60-binding peptide organizes fullerenes

**Figure 1:**
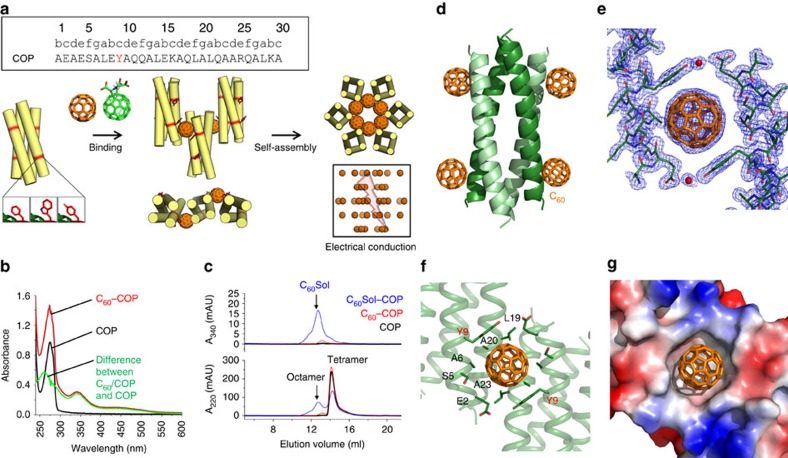
Protein/C_60_ super-assembly. (**a**) COP, a stable tetramer in isolation, interacts with C_60_ moieties by means of a surface-binding site that includes Tyr residues (other aromatic side chains also likely admissible), and further self-assembles into a co-crystalline array with fullerene. (**b**) Ultraviolet absorption spectra of a C_60_/COP suspension and COP alone demonstrate that primitive fullerene (green) dissolves in the aqueous phase in the presence of protein. (**c**) SEC traces of COP alone or in association with C_60_ or C_60_Sol. Top and bottom plots show absorbances at 340 and 220 nm, respectively. The lower-retention peaks arising due the addition of C_60_ or C_60_Sol are consistent with the molecular weight of a COP octamer (for example, dimer of tetramers; Supplementary Fig. 10). (**d**) Each COP tetramer in the C_60_Sol–COP crystal is associated with four fullerenes (one per chain), each fullerene being wedged between two adjacent COP tetramers, for an overall stoichiometry of two fullerenes for one COP tetramers. (**e**) Omit map (2*F*_o_−*F*_c_, contoured at 1.2*σ*) showing electron density of the C_60_ group (orange sticks) sandwiched via *π*–*π* stacking between Tyr residues from adjacent COPs. (**f**) Residues involved in C_60_ coordination are shown with sticks and labelled. (**g**) Surface representation of the C_60_ coordination site, coloured by relative *in vacuo* electrostatic potential (red to blue corresponds to negative-to-positive relative potentials).

As a candidate for organizing C_60_, we considered a peptide we had designed in a previous study (sequence in Fig. 1a), which forms an anti-parallel coiled-coil tetramer at μM concentrations (Protein Data Bank, PDB, entry 3S0R)^11^. Two key properties appear to make the peptide suitable for assembling C_60_. First, the single aromatic residue in its sequence, tyrosine at position 9, is exposed and available for potential C_60_ binding^23^,^27^. Second, the peptide appears to have an exceptionally low barrier to crystallization, forming X-ray diffraction quality crystals within hours. Inter-tetramer contacts within the resulting lattice are not extensive (Supplementary Fig. 1), suggesting that the ease of crystallization may be due to an innately low penalty for freezing out conformational degrees of freedom.

Upon sonication, C_60_ was readily solubilized in an aqueous solution of the peptide, hereafter referred to as COP (C_60_-organizing peptide), but not buffer-only solutions. The resulting C_60_–COP suspensions, stable after centrifugation for at least 3 months (not monitored thereafter), produced characteristic absorbance spectra revealing the presence of both protein and the fullerene (Fig. 1b). Size-exclusion chromatography (SEC) of COP alone was consistent with its tetrameric oligomerization state (black in Fig. 1c). On the other hand, upon the solubilization of C_60_, an additional peak appeared in the chromatogram, corresponding to a species of molecular mass approximately that of a dimer of tetramers (red in Fig. 1c; Supplementary Fig. 2). This suggests that the solubilization of C_60_ occurs in a structurally specific manner with a change in oligomerization state of COP.

Despite COP's propensity to crystallize, attempts to co-crystallize C_60_ with COP were not met with success. The C_60_–COP suspensions did form crystals, but these appeared to be devoid of fullerene. We reasoned that this could be due to an insufficient amount of solubilized C_60_, such that not all binding sites on COP would be saturated and the protein-only species would selectively crystallize. Indeed, a rough estimate based on the C_60_–COP ultraviolet spectrum (Fig. 1b) and molar absorptivity of C_60_ at 340 nm taken from water/poly(vinylpyrrolidone) suspensions^28^, suggests one molecule of C_60_ for ∼24 COP tetramers (see Methods). To address this issue, we produced solutions of COP with C_60_ pyrrolidine Tris-acid (C_60_Sol; Supplementary Fig. 3), a more water-soluble analogue of C_60_ (solubility 0.002–0.005 mg ml^−1^ at pH 7.4). The SEC chromatogram of the COP–C_60_Sol suspension again clearly shows two peaks—one corresponding to COP alone and another with apparent molecular weight corresponding to a dimer of COP tetramers (compare black and blue traces in Fig. 1c; also Supplementary Fig. 2). Further, absorbance at 340 nm (specific to the fullerene) clearly demonstrates that all of C_60_Sol elutes in the second (octameric) peak, arguing for a specific structure-based association (top plot, blue trace Fig. 1c). These results are further supported by analytical ultracentrifugation (AUC) sedimentation equilibrium experiments at a range of concentrations, showing tetramer–octamer equilibrium for C_60_Sol–COP solutions (with a dissociation constant of 118 μM), whereas a single-species monomer model is sufficient for COP alone (Supplementary Methods).

Crystals from the resulting suspension grew within 24 h in several conditions, and three separate structures of the C_60_Sol–COP complex were resolved to 1.67, 1.76 and 2.35 Å, respectively (Fig. 1d–g; Supplementary Figs 5–6; Table 1). To our knowledge, these represent first high-resolution structures of a protein–fullerene complex. As in the protein-only structure, COP forms a canonical tetrameric anti-parallel coiled coil^29^. Each tetramer presents four tyrosine residues (one per monomer) in exterior **c** positions of the coiled-coil heptad, and each of these engages a C_60_ moiety. One C_60_ is wedged between two Tyr residues donated by adjacent tetramers, such that two tetramers are needed to coordinate one C_60_ (Fig. 1f). This arrangement fits well with the apparent octameric peak in the SEC chromatogram and AUC profiles of C_60_Sol–COP (Fig. 1c; Supplementary Fig. 4), suggesting that higher-order organization begins already in solution before crystallization. The water-solubilizing Tris-acid side chain of C_60_Sol is not visible in the electron density map. The group likely points into the solvated inner channel of the crystal and is highly mobile as the C_60_ core rapidly rotates around its centre. This is consistent with the intended role of the side chain, to increasing the solubility of the fullerene, whereas the C_60_ core appears responsible for the specific packing arrangement.

**Figure 2:**
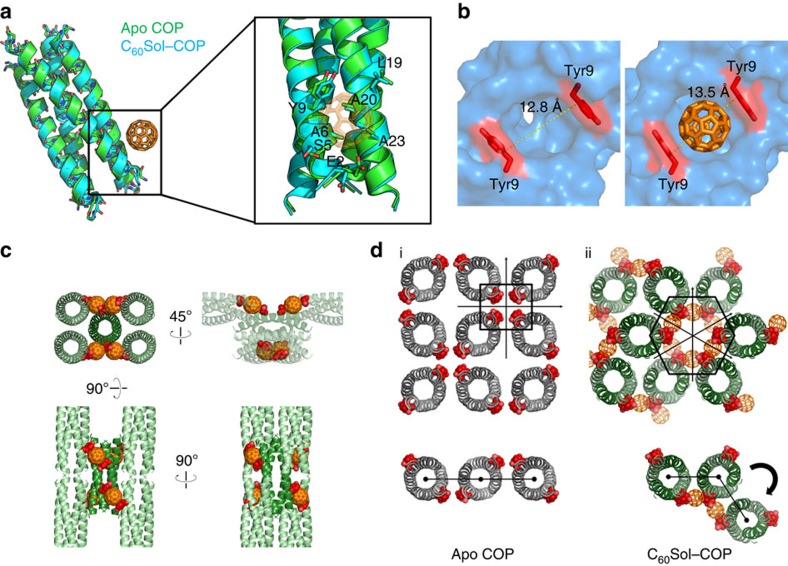
COP crystal adjusts to incorporate fullerene. (**a**) Superposition of *apo* COP (green) and C_60_Sol–COP (cyan) shows no significant structural changes in the helix bundle. Side-chain differences around the fullerene-binding site are highlighted in the box. (**b**) The distance between aromatic Tyr9 residues adjusts in C_60_Sol–COP to incorporate the fullerene. (**c**) Different views of the COP–fullerene lattice. (**d**) Significant changes in the crystal lattice between COP alone and C_60_Sol–COP structures. Viewed from the top, C_60_Sol–COP forms a honeycomb structure (ii), whereas *apo* COP exhibits a tetrameric cube pattern (i).

The interaction between COP and the fullerene group involves non-polar contacts (Supplementary Fig. 7a), with C_60_ fitting perfectly into a symmetric hydrophobic cavity created by helices of two adjacent COP tetramers. The dominant contact appears to be the *π*–*π* aromatic stacking between C_60_ and Tyr9, donated by each tetramer, while several aliphatic carbons line the pocket to further support the bound pose (Supplementary Fig. 7a; Fig. 1f,g). Interestingly, the conformation of COP itself is largely unchanged between the protein-only and protein/fullerene structures (Fig. 2a). Indeed, the Cα root mean squared deviation (r.m.s.d.) between the two tetramers is only 0.68 Å (over 120 atoms; Fig. 2a,b), the full-backbone r.m.s.d. is 0.75 Å (over 600 atoms), and the two structures represent coiled coils with essentially identical Crick parameters^30^ (see Methods; Supplementary Table 1). On the other hand, there is considerable difference in the arrangement of COP tetramers in the corresponding crystals: COP alone assembles into a body-centered cube, while C_60_Sol–COP has a honeycomb structure with parallel hexameric channels (Fig. 2c,d). This, together with the fact that neither of the crystals exhibit what would appear to be strong protein–protein interactions between tetramers, argues that the C_60_ group is chiefly responsible for driving the assembly of COP tetramers into the pattern observed in the co-crystal. In fact, in the fullerene bound structure, no contacts between adjacent COP tetramers occur outside of the C_60_-binding site. Furthermore, the three different COP–C_60_Sol co-crystals obtained under different conditions (see Methods) all produced effectively identical binding interfaces and assembly geometries, arguing that fullerene may have a strong preference for the observed coordination geometry.

### Helix–helix motif expected to bind fullerene tightly

We next ask whether the helix(Tyr)–C_60_–helix(Tyr)-binding mode could serve as a general co-organizer of proteins and fullerene. A necessary (but not sufficient) condition for this is that the motif would need to provide sufficient binding energy to drive assembly into a variety of desired arrangement. So we sought to quantify the affinity of C_60_ for the identified binding site. Direct equilibrium measurement of C_60_–protein association is complicated by the exceedingly low solubility of C_60_ in aqueous solution. Even the C_60_Sol derivative has limited water solubility, hampering binding studies. We thus turned to explicit-solvent molecular dynamics simulations to characterize the strength of COP–C_60_ association. The observed binding mode is a ternary interaction between two COP tetramers and one fullerene. To simplify the analysis, we concentrated on one half site of the symmetric binding pocket, looking to characterize the affinity of one C_60_ for one COP. Using the crystal structure as the starting bound configuration, thermodynamics of binding was characterized using a modification of the double-decoupling method^31^ in conjunction with the free energy perturbation (FEP) approach^32^ (see Methods; Supplementary Fig. 8). A total simulation time of 336 ns permitted accurate monitoring of convergence, with the standard-state free energy of C_60_–COP binding estimated at −9.8±0.3 kcal mol^−1^. This corresponds to a dissociation constant in the range of 40–100 nM, confirming the suspicion that C_60_ binding provides substantial energy to drive the assembly of COP units. In fact, the true energetic contribution of C_60_ is likely even larger as some positive cooperativity between the two motif half sites would be expected due to direct (albeit not extensive) favourable protein–protein interactions. Interestingly, we find that the *π*–*π* stacking between C_60_ and Tyr9 is not sufficient to explain the strong interaction, as the affinities of C_60_ for an isolated Tyr residue (acetylated and methylamidated at the N- and C termini, respectively) or a Tyr side-chain analogue (*p*-methylphenol) are estimated to be in the mM range (Supplementary Fig. 8b,c). Thus, additional aliphatic contacts in the binding pocket are essential for the collective binding mode and the high affinity.

### Fullerene-binding motif composed of designable elements

Another necessary property of a generic protein–fullerene organizing motif is that it must be ‘designable' in the context of a multitude of protein lattices—that is, the required geometry should be easily achievable with natural amino-acid sequences. Using the structural search engine MASTER^33^, we found that all of the interfaces involved in the motif are indeed highly abundant in nature (and are thus necessarily designable), with emergent sequence preferences in agreement with the corresponding region of COP (Methods; Supplementary Fig. 7b). Further, even the entire binding motif, composed of four disjoint helical segments that account for all contacts with C_60_ in the supercrystal, has precedence in PDB lattices. In fact, within a homology/redundancy-pruned subset of the PDB (13,400 entries), we found 180 unique instances of matching geometries (below 1.9 Å backbone r.m.s.d., computed over 112 atoms) within 21 unique lattices (Supplementary Fig. 7c). That is, ∼0.15% of proteins in the PDB already exhibit backbone geometries similar to the one housing a bound C_60_ in the co-crystal, suggesting that it may be possible to engineer a variety of fullerene/protein co-assemblies by perturbing sequences of existing parent proteins. Supplementary Fig. 7c shows examples of such putative co-assemblies, where C_60_ is computationally placed into existing lattices matching the binding motif, giving diverse C_60_-to-C_60_ distances and lattice arrangements. Of course, the design of such assemblies will involve not only the placement of a C_60_-binding motif, but also any appropriate accommodating changes to surrounding amino acids. Further, there is no guarantee that the crystal form will not change upon these perturbations. Still, that our identified motif appears ‘canonical' in terms of its constituent protein–protein interfaces is encouraging for future design applications.

### Fullerene–protein crystal has emergent electronic properties

**Figure 3:**
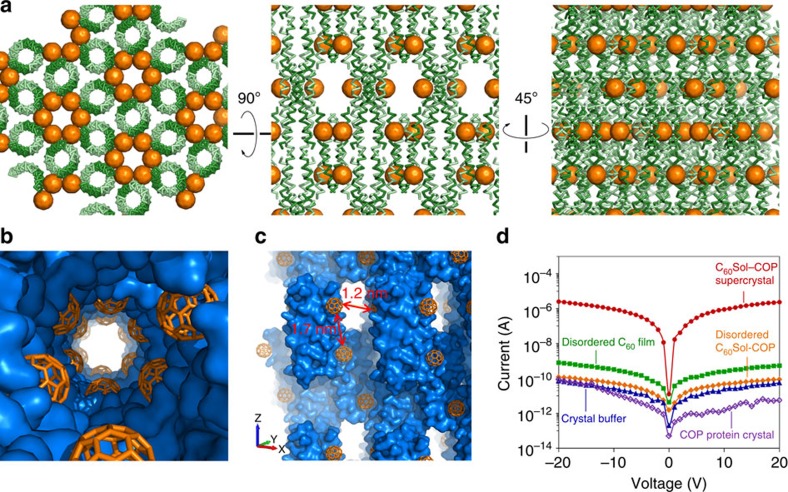
Assembly of fullerenes endows crystal with electronic transport capabilities. (**a**) Three views of the C_60_Sol–COP crystal lattice. (**b**) C_60_ groups are arranged in a helical manner along parallel inner channels in the assembly. (**c**) A side view of the channel showing nearest-neighbor inter-C_60_ distances. (**d**) Semi-logarithmic current–voltage characteristic of C_60_Sol–COP supercrystal (red dots) and disordered C_60_Sol–COP (orange diamonds). Disordered C_60_ film dried from a bare C_60_/toluene solution (green squares), crystal buffer solution (blue triangles) and a COP-alone protein crystal (violet open circles) were also characterized as controls.

The honeycomb structure of C_60_Sol–COP is intriguing from the perspective of its electronic properties. Within the helical arrangement of fullerenes, inter-C_60_ distances appear sufficiently close for potential long-range electronic transfer, especially given the organized nature of the surroundings^34^,^35^ (Fig. 3a–c). For this reason, we sought to characterize the electrical conductance of the co-crystal. Current–voltage (*I*–*V*) characteristic of disordered C_60_ films showed high electrical resistance of 2.24 × 10^11^ Ω (Fig. 3d and Supplementary Fig. 9). In addition, COP-alone crystals or crystal buffer similarly showed high resistance, with only 5–10 pA of currents measured with up to 20 V of voltage sweep. On the other hand, C_60_Sol–COP supercrystals (of similar dimension as protein-alone crystals) exhibited high electrical conductance (1.40 × 10^−7^ S, corresponding to resistance of 7.14 × 10^6^ Ω) with at least four orders of magnitude higher currents than in any of the controls (Fig. 3d). We speculate that the periodic arrangement of fullerene groups in the co-crystal may facilitate electron wave delocalization over the assembly. This would promote coherent electron transport through the structure with the carrier mobility expected to be several orders of magnitude higher than in disordered systems characterized by hopping transport^36^. Inter-fullerene nearest-neighbour distances in the C_60_Sol–COP supercrystal alternate between 1.2 and 1.7 nm (Fig. 3c). For comparison, strong electron wave delocalization was previously observed when the nearest-neighbour distance approached ∼1.5 nm in one-dimensional fullerene wires^37^. An alternative explanation of the observed conductive property is that the hexameric channels in the co-crystal may contain unattached/disordered fullerene moieties that are free to diffuse in the channel and can shuttle electrons between ordered in-lattice fullerenes. In either case, as shown in Fig. 3d (yellow dots), destruction of crystalline order (by placement in vacuum) results in very high electrical resistance. In fact, the current measured here is even lower than that of the disordered C_60_ film. This indicates that the high conductivity of the C_60_Sol–COP supercrystal is not a trivial property of crystal dimension and/or molecular composition, but rather originates from specific electronic coupling/delocalization in the assembly.

## Discussion

The aim of programmable self-assembly is to anticipate and harness unique collective properties that arise from precisely organized molecular building blocks. To this end, achieving atomic-level precision is crucial. This work demonstrates the first atomic resolution structures of a fullerene–protein assembly, establishing the feasibility of creating such objects, and further suggests a possible design principle for engineering such assemblies in general. How robust the discovered C_60_-binding motif is towards designing novel assemblies will need to be tested through a number of future design studies. However, the straightforward manner in which self-organization arose in our case, the simplicity of the C_60_-organizing motif in the lattice, together with its high affinity and the ubiquity of associated interfaces in natural protein lattices, are certainly promising with respect to the general applicability of the design principle. Our work also demonstrates the potential utility of exploring C_60_/protein co-organization, as derived supercrystals already showed synergistic charge conductance properties. Taken together, these results point to an exciting direction of inquiry towards generating protein–fullerene assemblies for the study and design of novel properties.

## Methods

### Peptide synthesis and purification

Peptides were synthesized by CEM Discover microwave synthesizer using Fmoc chemistry at 100-μmol scales. The Fmoc protecting group was removed by piperidine/dimethylformamide solution (20/80 v/v); at each coupling step reactants were added with the amino acid:HBTU:DIEA:resin ratio of 5:4.9:10:1. Products were cleaved from the H-Rink Amide-ChemMatrix (PCAS, 0.53 mmol g^−1^ loading) in a cleavage cocktail solution (trifluoroacetic acid (TFA)/triisopropyl silane/deionized water, 95/2.5/2.5 v/v) for 2 h and the remaining solution was vapourized with N_2_ gas. Peptide was precipitated with cold diethyl ether (Aldrich) and dried in vacuum. After dissolving the peptides in DI H2O, purification proceeded by preparative reverse-phase high-performance liquid chromatography (Waters prep 150 LC System) using preparative C4 column (XBridge BEH300 Prep C4 5 um) and a linear gradient of buffer A (99.9% H2O and 0.1 % TFA) and buffer B (90% acetonitrile, 9.9% H2O and 0.1 % TFA). Molecular mass of the peptide was confirmed by matrix-assisted laser desorption/ionization-time of flight mass spectrometry (Bruker Ultraflex III). Products had over 95 % purity.

### Preparation of peptide/fullerene solutions

Samples were prepared with 8 mg ml^−1^ protein solution (COP) in 25 mM Tris pH 8.0 buffer solution and 1 mg C_60_ or C_60_ pyrrolidine Tris-acid (Aldrich). Fullerene powder was mixed with pre-made 0.2 ml of 8 mg ml^−1^ protein solution in 25-mM Tris pH 8.0. The sample was then tip-sonicated (QSonica, Q125, 1/8th inch tip) on an ice bath for 5 min to be saturated of fullerene. Ice-bath cooling was to prevent excessive sample heating and destabilization of protein structure. The sonicated samples were warmed up to room temperature and centrifuged at 14,500*g* for 10 min (Eppendorf, Centrifuge 5430R).

### Ultraviolet–visible absorption spectroscopy

Ultraviolet absorption spectra of the COP alone and C_60_/COP were recorded using a Hewlett Packard 8453 spectrometer in 1 cm Hellma Quartz SUPRASIL (QS) cells. The COP and C_60_/COP were prepared in a buffer of 20 mM sodium phosphate, 100 mM NaCl and pH 7.5. Ultraviolet–visible spectra of C_60_/COP and COP were used to roughly estimate the concentration of solubilized fullerene by absorbance at 340 nm (the molar absorptivity of 49,000 M^−1^ cm^−1^ was used for C_60_, (ref. 28). The resulting molar concentration of solubilized C_60_ in the C_60_/COP solution was 6.22 μM (compared with COP at 585 μM in the same solution).

### Size-exclusion chromatography

Size-exclusive gel filtration elution profiles were obtained using a Superdex 75 10/300 GL column with a GE Healthcare fast performance liquid chromatography (FPLC) system (Amersham Pharmacia Biosystems). Peptides (at 200 μM) were prepared in a buffer of 20 mM sodium phosphate, 100 mM NaCl and pH 7.5 at room temperature. 200 μl of each sample were loaded and eluted with the same buffer. The column was equilibrated in 20 mM sodium phosphate, 100 mM NaCl and pH 7.5 with a mobile phase flow rate of 0.5 ml min^−1^, and absorbance at 220, 280 and 340 nm was recorded. Calibration curves were obtained using the molecular-weight standard kit, MWGF70 6,500–66,000 (Supplementary Fig. 10).

### Analytical ultracentrifugation

Oligomerization states of COP and C_60_Sol–COP were determined by equilibrium sedimentation performed at 25 °C using a Beckman XL-I analytical ultracentrifuge. Both solutions were prepared in a buffer of 25 mM Tris pH 8.0. Equilibrium radial concentration gradients at four different rotor speeds (25, 30, 35 and 40 K r.p.m.) were acquired as absorbance scans at 340 nm for C_60_Sol with COP and 280 nm for COP peptide alone. Data were globally fit to single-species or two-species models of equilibrium sedimentation by a nonlinear least-squares method using IGOR Pro (Wavemetrics), and the best-fitting model was accepted^38^. Supplementary Figure 4 shows sedimentation equilibrium profiles of C_60_Sol–COP along with corresponding species distribution plots consistent with a tetramer–octamer equilibrium, whereas COP alone appears as a tight tetramer. This is consistent with results from SEC, shown in Fig. 1c and Supplementary Fig. 2.

### Crystallization, data collection and processing

The first X-ray diffraction quality crystal (C_60_Sol–COP-1) was obtained by the hanging-drop vapour diffusion technique at 291 K, over a period of 15 days in a 2 μl drop consisting of 1:1 v/v mixture of 1 mgml^−1^ protein solution in 20 mM sodium phosphate/100 mM NaCl pH 7.5 buffer and a reservoir solution of 17 mM lithium sulfate monohydrate, 85 mM Tris-hydrochloride sodium pH 8.5, 25.5% polyethylene glycol (PEG) 4,000, 25% v/v glycerol (Hampton Research sparse matrix). The crystal was flash-frozen, and diffraction data were collected at the 24-ID-E NE-CAT beamline at the Argonne National Laboratory. Data sets were indexed and integrated with MOSFLM^39^,^40^, and scaled using SCALA3 (Collaborative Computational Project, Number 4, 1994)^41^. Diffraction data were recorded to a maximum resolution of 2.35 Å (Table 1).

Subsequent crystallization attempts were performed with higher concentrations of the C_60_Sol/COP suspension, using commercially available sparse-matrix screens from Hampton Research and the hanging-drop vapour diffusion method at 295 K. Diffraction-quality crystals of C_60_Sol–COP (C_60_Sol–COP-3) were obtained by mixing equal volumes of the C_60_Sol–COP mixture at 8 mg ml^−1^ in 25 mM Tris pH 8.0 and reservoir solution consisting of 0.2 M ammonium acetate, 0.1 M sodium citrate tribasic dihydrate pH 5.6, 30% w/v polyethylene glycol 4,000. Microcrystals grew within 24 h, with larger oval-shaped crystals appearing in several days (Supplementary Fig. 5). Crystals were cryoprotected using reservoir solution supplemented with an additional 30% (v/v) glycerol and were flash-cooled in liquid nitrogen. Diffraction data, extending to 1.67 Å resolution, were collected at 100 K on beamline 7A equipped with an ADSC Quantum 270 CCD detector at Pohang Accelerator Laboratory (PAL, Pohang, Korea). The C_60_Sol–COP complex crystal belonged to space group *P*6_2_, with unit cell parameters *a*=*b*=42.1, *c*=66.7 Å, *α*=*β*=90.0 and *γ*=120.0°. Data were processed and scaled using the programs DENZO and SCALEPACK from the HKL-2000 program suite^42^. The Matthews coefficient^43^ for C_60_Sol–COP was 2.54 Å^3^ Da^−1^ and the estimated solvent content was 51.5%; there were two COP molecules and one C_60_Sol in an asymmetric unit.

In addition to the above, diffraction-quality crystals were also obtained in three other conditions (1.5 M ammonium sulfate, 0.1 M Tris pH 8.5, 12% v/v glycerol; 0.1 M HEPES–Na pH 7.5, 0.8 M potassium sodium tartrate tetrahydrate; and 0.1 M *N*-(2-acetamido)iminodiacetic acid, *N*-(carbamoylmethyl)iminodiacetic acid (ADA) buffer pH 6.5, 1 M ammonium phosphate dibasic), in each case yielding identical unit cell and space group, thus showing the same assembly geometry. Crystals grown under the latter condition diffracted to 1.76 Å at a home source (C_60_Sol–COP-2).

### Structure solution and refinement

For all the data sets, structure determination was carried out by molecular replacement using the programme PHASER^44^. The Matthews coefficient suggested a dimeric helix in the asymmetric unit. Molecular replacement calculations were performed using the dimeric unit of a polyalanine model obtained from coordinates of previously solved crystal structure 3S0R as the search probe. The solution model was subjected for rigid body refinement followed by iterative model building and restrained refinement protocols implemented in Auto Build module of PHENIX^45^. All side chains were traced in the electron density map. During the course of map tracing, electron density for fullerene was clearly visible and modelled for refinement.

During data analysis, it was found that the crystal (C_60_Sol–COP-1) was merohedrally twinned. The H-test results, |*H*|=0.024 (0.50 for untwinned and 0.0 for 50% twinned) and *H*^2^=0.001 (0.33 for untwinned and 0.0 for 50% twinned), were indicative of merohedral twinning with the twin law (h, -h-k, -l), where *H*=|*I*_1_−*I*_2_|/|*I*_1_+*I*_2_|, *I*_1_ and *I*_2_ are twin-related acentric reflections. The cumulative distribution of H^46^,^47^ and Britton plots^48^,^49^ estimated twin domain fraction (*α*) to be 0.478 and 0.447, respectively.

As the estimated twin fraction was close to 0.5, the data were not detwinned for further refinement. Instead, the refinement was carried out by refining both parameters of the model and twin fraction. The PHENIX^45^ refinement protocol, which implements this option, was used.

The PHENIX refinement protocol was used. Upon converging, the refinement strategy produced model with good *R*_work_/*R*_free_ statistics in each case (Table 1). Model building was further carried out manually using COOT^50^. Structure figures were created using the programme PyMOL (Schrödinger, LLC). Crystallographic data statistics are summarized in Table 1.

### Coiled-coil parameter fitting

Parameter fits were performed using CCCP ( http://grigoryanlab.org/cccp) via the ‘global symmetric' fit option, where ideal symmetry (in this case D2) is assumed^30^. The *apo* and C_60_-bound structures fit within 0.6 and 0.4 Å, respectively, indicating that they both closely resembled an ideal coiled coil. Key parameters are listed in Supplementary Table 1. Detailed parameter definitions and information on the fitting procedure can be found in reference^30^.

### Binding free-energy calculation

The NAMD 2.10 software package, developed by the Theoretical and Computational Biophysics Group in the Beckman Institute for Advanced Science and Technology at the University of Illinois at Urbana-Champaign^51^, in conjunction with CHARMM22 force field^52^ was used for this study. A new atom type was created for the C_60_ carbon (CA60), which was identical to the aromatic carbon atom type in CHARMM22 (type CA) in all aspects except for the equilibrium CA60–CA60 bond length, which was set to 1.4392 Å to match the experimentally observed average bond length in C_60_ (ref. 53). All simulations were performed in explicit TIP3P water; a padding of 8 Å was used for initial solvation, with sodium/chloride counterions added to achieve charge neutrality as necessary. Periodic boundary conditions were applied and all simulations were performed in the NTP ensemble at 298.15 K and 1 atm. Explicit calculation of long-range interactions was cutoff at 10 Å, with a switching function starting at 6 Å. Particle Mesh Ewald method was used to correct for long-range electrostatics^54^ and an analytical correction was used to capture long-range van der Waals interactions^55^. Pande and co-workers have shown that with these corrections, the 6/10 Å non-bond cutoff schedule performed as well as longer cutoffs in free energy of solvation calculations^56^.

To compute the free energy of C_60_–COP association, we followed the double-decoupling framework outlined by McCammon and co-workers^31^. In this approach, one seeks to compute the free energy of decoupling the ligand (here C_60_) from the rest of the system when it is either bound to the receptor (here COP) or solvated by itself. The standard-state free energy of binding is then related to the difference between the two decoupling free energies, appropriately corrected for the standard-state concentration^31^. We sought to use the method of FEP to compute individual decoupling free energies, but the direct application of the method to C_60_ exhibited very strong hysteresis between forward and reverse simulations (that is, decoupling C_60_ and coupling it back, respectively). Because C_60_ is hollow, with enough space inside for several water molecules, as the molecule is decoupled, water rushes in to occupy the available space. However, during the reverse simulation, as C_60_ is coupled back to the system, water molecules tend to remain trapped inside the fullerene, leading to a very different end state. Note that use of the soft-core van der Waals scaling^55^, implemented in NAMD, does not resolve this issue as there is little to encourage water molecules to escape the core of fullerene as it is coupled back. This very large hysteresis meant that we could not claim good convergence (and hence accuracy) of either forward or reverse simulations.

To resolve this issue, we introduced an intermediate step in the C_60_ decoupling/coupling transformation, designed to provide reversibility, slightly adjusting the double-decoupling framework. The idea was to introduce an artificial atom, with a size to roughly match the radius of C_60_, which could be used to ‘make room' for C_60_ before the molecule is coupled to the system. A new atom type was created, called C60D (for C_60_ ‘dummy'), with a van der Waals radius of 4.5 Å and a Lennard-Jones well depth of −1.0 kcal mol^−1^. Because C60D is a single atom, and not hollow-like C_60_, the soft-core van der Waals potential will indeed gradually repel water molecules as C60D appears. Thus, the two decoupling transformations were altered as follows:


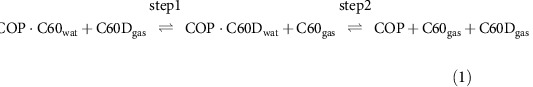



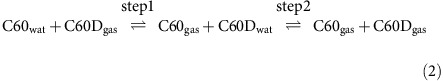


where subscripts wat and gas indicate that the corresponding molecule is either fully coupled to the system (that is, in water) or fully decoupled from it (that is, in the gas phase), respectively. The initial state of transformation 1 involves the complex between COP and C_60_ (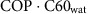) and a decoupled C60D (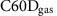) overlapping the fullerene. The first step of the transformation involves decoupling C_60_ from the system, while C60D is coupled, such that the intermediate step has C_60_ in the gas phase (C60D_gas_) while C60D is fully interacting with the system, occupying the fullerene-binding site (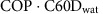). The second step of the transformation then decouples C60D as well, such that the end state involves COP in solvent alone with both C_60_ and C60D in the gas phase. Because gaseous C60D is present in both end states, its contribution to the total free-energy difference cancels, such that the net transformation still represents just the decoupling of C_60_. On the other hand, the presence of C60D and the intermediate state address the reversibility of the transformation. Because the first step in the reverse direction involves coupling of C60D, room is created in the solvent before C_60_ is reintroduced and C60D is once again decoupled in the second step. To prevent C60D from diffusing away from the binding site at any point in the simulation, harmonic restraints were applied between C60D, and Cγ, Cɛ_1_ and Cɛ_2_ atoms of the binding site Tyr (residue 9), with equilibrium distances of 6.7, 6.7 and 7.0 Å, respectively (taken from the crystal structure by initially placing C60D in the geometric centre of the bound C_60_), and a force constant of 10 kcal mol^−1^ Å^−2^. Note that these restraints do not contribute to the FEP calculation (since their energy is independent of the coupling parameter) and their presence fully cancels between end states of the transformation. Another restraint was needed to make sure C_60_ does not diffuse far from the binding site when decoupled, which would create convergence difficulties. A harmonic restraint was applied between the centre of mass of C_60_ and C60D, with an equilibrium distance of zero and a force constant that increased from 0 to 10 kcal mol^−1^ Å^−2^ as C_60_ was decoupled from the system. Specifically, if *λ* is the FEP coupling parameter for the current window (with 0 and 1 corresponding to C_60_ being fully coupled and decoupled, respectively), the force constant used was 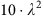 kcal mol^−1^ Å^−2^. The energy of this restraint was accounted for in FEP calculations, so that the final free-energy change for transformation 1 represented the difference between a state where C_60_ is fully coupled and bound to COP and one where C_60_ is decoupled from the system, but harmonically restrained to remain in the vicinity of the binding site. To remove the influence of this restraint and correct for the standard state, this free-energy change was corrected by 
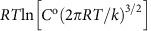
, where 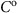 is the standard-state concentration and *k* is the force constant of the C_60_ restraint in the decoupled state (that is, 10 kcal mol^−1^ Å^−2^)^31^,^57^.

Transformation 2 is similar to transformation 1, but with no protein. In the first step, C_60_ is decoupled from solvent as C60D is coupled, whereas the second step decouples C60D. As with transformation 1, the influence of C60D cancels between the two end states, with the total free-energy difference corresponding to that of decoupling C_60_ from solvent. However, the intermediate step again renders the path reversible. As with transformation 1, here it was important for C_60_ and C60D to be approximately coincident throughout the simulation (so that, for example, in the first step of the reverse simulation coupling of C60D creates a cavity in the right location within the solvent for C_60_ to couple into later). For this reason, a harmonic restraint was introduced between the centroid of C_60_ and C60D, with equilibrium distance of zero and a force constant of 10 kcal mol^−1^ Å^−2^. Note that the contribution of this constant restraint cancels between the two end states (so the total free-energy change of transformation 2 is still that of decoupling C_60_ alone), and its energy does not influence FEP calculations.

Since C_60_ remains decoupled (and restrained to C60D) throughout step 2 of both transformations, it does not contribute to the free-energy change associated with these steps. For this reason, C_60_ need not be explicitly present in simulations of these steps and was omitted for simplicity.

*FEP details and results*. NAMD's alchemical transformation module (in conjunction with the FEP method) and the collective variable module (for introduction of restraints) were used to implement the above transformations. The soft-core van der Waals radius-shifting coefficient (parameter alchVdwShiftCoeff) was set to 8 Å^2^ in the first step of both transformations and to 20 Å^2^ in the second step of both transformations (values were chosen to produce smooth transitions in short FEP test runs). All four steps were carried out using 20 FEP windows, with the coupling parameter varying uniformly from 0 to 1. Each window involved 10 ps of equilibration followed by 190 ps of data collection. At the start of each simulation, the system (upon being minimized for 1,000 steps) was pre-equilibrated for 200 ps. Each step of both transformations was run 10 times in both forward and reverse directions, using different random seeds. Thus, a total of 336 ns of simulation was performed. The final results are summarized in Supplementary Fig. 8a, where values for reverse transformations have been negated to represent free energies in the decoupling direction. Error bars represent s.e.'s of the cumulative free-energy difference, computed over the 10 simulations run for each step/direction combination. Clearly, all steps exhibit excellent convergence and reversibility. The standard-state free energy of C60–COP binding was computed as:


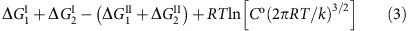


where 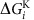 is the free-energy change of the *i*th step of transformation *K*. The final estimate amounted to −9.8±0.3 kcal mol^−1^, where the uncertainty was calculated by error propagation using s.e.'s emergent from combining all simulations of each step (both forward and reverse).

### Association of fullerene with individual aromatic groups

An analogous approach was also used to calculate the affinity of C_60_ for a disembodied Tyr residue (acetylated and methyl-amidated on the N- and C termini, respectively) and a Tyr side-chain analogue (*p*-methylphenol). The only difference was that in these cases an additional constant harmonic restraint, between the centre of mass of C_60_ and C60D, was added throughout step 1 of transformation 1. This restraint, with a force constant of 1.0 kcal mol^−1^ Å^−2^ and equilibrium distance of 0 Å, prevented C_60_ from dissociating from the bound molecule in the initial FEP window, which otherwise occasionally occurred in some trajectories and limited the amount of useful sampling. The effect of this restraint was removed from the final estimate by applying the standard importance sampling formula^58^ to adjust the expectation computed in FEP^59^. The final standard-state binding free-energy estimates were −1.76±0.15 for C_60_ and isolated Tyr, and −1.53±0.07 for C_60_ and *p*-methylphenol (Supplementary Fig. 8b–c). These correspond to dissociation constants in the mM range, meaning that the affinity is expected to be extremely weak.

### Designability analysis

To estimate the natural abundance of structural motifs surrounding the C_60_-binding site, search engine MASTER^33^ (grigoryanlab.org/master) was used to search a highly non-redundant subset of the PDB. Specifically, the weekly BLASTclust-based clustering^60^ of all PDB chains was downloaded on 22 October 2014, and the first chain from each cluster selected, filtering for X-ray structures resolved to 3 Å or below. The asymmetric unit of each of the entries was then downloaded and the crystallographic lattice generated, keeping all images that were reasonably close to the initial unit (defined as having at least three atoms within 16 Å of any atom in the initial unit). The resulting lattices were then combined into a MASTER database of 13,400 entries. All searches were performed using the full-backbone setting of MASTER, which provably finds the closest matches to the query in terms of the heavy-atom backbone r.m.s.d. (that is, N, CA, C and O). The full C_60_-binding motif was defined as residues 2–9 on one pair of chains and 19–24 on the opposing pair, with individual interfaces of this motif defined accordingly (Supplementary Fig. 7b). Sequence logos in Supplementary Fig. 7b were generated by considering all matches within 0.3 Å and discarding those with identical sequences (although the database is highly non-redundant, matches of identical sequence are still possible when multiple-matching instances are found within the same lattice).

### Measurement of electrical conductance

Current versus voltage curves were obtained using the variable temperature microprobe system from MMR technologies coupled with HP 4145B semiconductor parameter analyser. The samples were deposited on a degenerately doped silicon substrate with 200The thermal oxide, which was photolithographically pre-patterned with Au/Cr (45 nm/5 nm) electrodes. The channel length and width were 10 and 6,000 μm, respectively.

## Additional information

**Accession codes:** The coordinates for the X-ray structures have been deposited to the Protein Data Bank (PDB) with accession codes 5ET3, 5HKN and 5HKR.

**How to cite this article:** Kim, K.-H. *et al*. Protein-directed self-assembly of a fullerene crystal. *Nat. Commun.* 7:11429 doi: 10.1038/ncomms11429 (2016).

## Supplementary Material

Supplementary Information Supplementary Figures 1-10, Supplementary Table 1 and Supplementary References.

## Figures and Tables

**Table 1:** Statistics on data collection and refinement of C_60_Sol–COP complex.

Data set:*	**C**_**60**_**Sol–COP-1**	**C**_**60**_**Sol–COP-2**	**C**_**60**_**Sol–COP-3**
Crystallization conditions	17 mM LiSO_4_85 mM Tris-HCl25.5% PEG 4,000pH 8.5	0.1 M ADA1 M NH_4_H_2_PO_4_pH 6.5	0.2 M CH_3_CO_2_NH_4_0.1 M HOC(COONa) (CH_2_COONa)_2_·2H_2_O 30% PEG 4,000pH 5.6
Data collection statistics			
Beam line	24IDE,NE-CAT	Home source†	PLS,BL-7A
Wavelength (Å)	0.97919	1.54178	1.00000
Space group	*P*6_2_	*P*6_2_	*P*6_2_
Cell dimensions			
*a*, *b*, *c* (Å)	41.71, 41.71, 66.81	41.71, 41.71, 67.23	42,15, 42.15, 66.79
*α*, *β*, *γ* (°)	90, 90, 120	90, 90, 120	90, 90, 120
Resolution (Å)‡	50.0–2.35 (2.48–2.35)	50.0–1.76 (1.86–1.76)	50–1.67 (1.73–1.67)
*R*_merge_	0.135 (0.709)	0.050 (0.309)	0.066 (0.188)
*I*/*σ*(*I*)	15.3 (4.2)	15.6 (2.4)	30.9 (10.9)
Completeness (%)	100 (100)	94.6 (69.1)	95.9 (99.7)
Multiplicity	13.7 (14.1)	5.1 (1.8)	16.3 (11.3)
Total/unique reflections	38460/2799	31977/6255	336749/7862
			
Refinement statistics			
Resolution (Å)	36.13–2.35	24.61–1.76	15.0–1.67
Number of reflections	5421	6235	7489
Twin fraction (*α*), estimated and refined	0.478 (S(H) plot), 0.447 (Britton plot), 0.5 (refined)		
*R*_work_/*R*_free_	0.2019/0.2338	0.2027/0.2391	0.2181/0.2444
Number of atoms	512	526	535
Proteins	446	446	446
Ligand/ion	60	60	60
Water	6	20	29
B-factors (Å^2^)			
Average B-factors (Å^2^)	30.8	27.0	22.5
Proteins	30.6	26.3	21.3
Ligand/ion	31.8	29.5	27.5
Waters	30.6	34.4	30.5
r.m.s.d.'s			
Bond lengths (Å)	0.007	0.007	0.008
Bond angles (°)	0.808	0.828	0.893
Ramachandran regions (%)			
Most favourable:	100	100	100
Additional allowed	0.0	0.0	0.0
Generously allowed	0.0	0.0	0.0

r.m.s.d.'s, root mean squared deviations.

*R*_work_=Σ||*F*_obs_|−|*F*_calc_||/Σ|*F*_obs_|, where *F*_obs_ and *F*_calc_ are calculated observed and calculated structure factor amplitudes, respectively, *R*_free_ was calculated as *R*_work_ using 10.0% of the randomly selected unique reflections that were not included in structure refinement.

^*^Structures of the same complex determined under different condition in different resolutions.

^†^Home source, CCMB (Center for Cellular and Molecular Biology), Hyderabad, India.

^‡^Highest resolution shell is shown in parenthesis.
