# Supplementary material for: Protein-directed self-assembly of a fullerene crystal
Source: Nat Commun. 2016 Apr 26;7:11429. doi: 10.1038/ncomms11429 (PMC4853425; doi:10.1038/ncomms11429)
Supplement: Supplementary Information — Supplementary Figures 1-10, Supplementary Table 1 and Supplementary References. [file ncomms11429-s1.pdf]

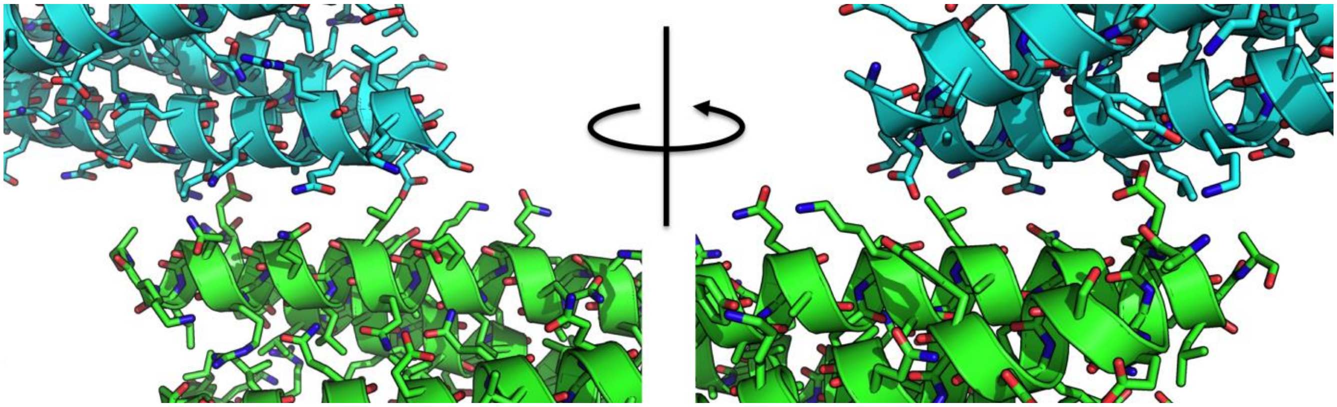

Supplementary Figure 1 | Crystal contacts in COP *apo* structure (PDB code 3S0R) Shown in cyan and green are two adjacent tetramers from the crystallographic lattice of COP, forming the only unique inter-tetramer interface in this body-centered cube structure. As can be seen, the interface does not appear to represent a strong interaction, lacking tight packing and significant hydrophobic surface area burial.

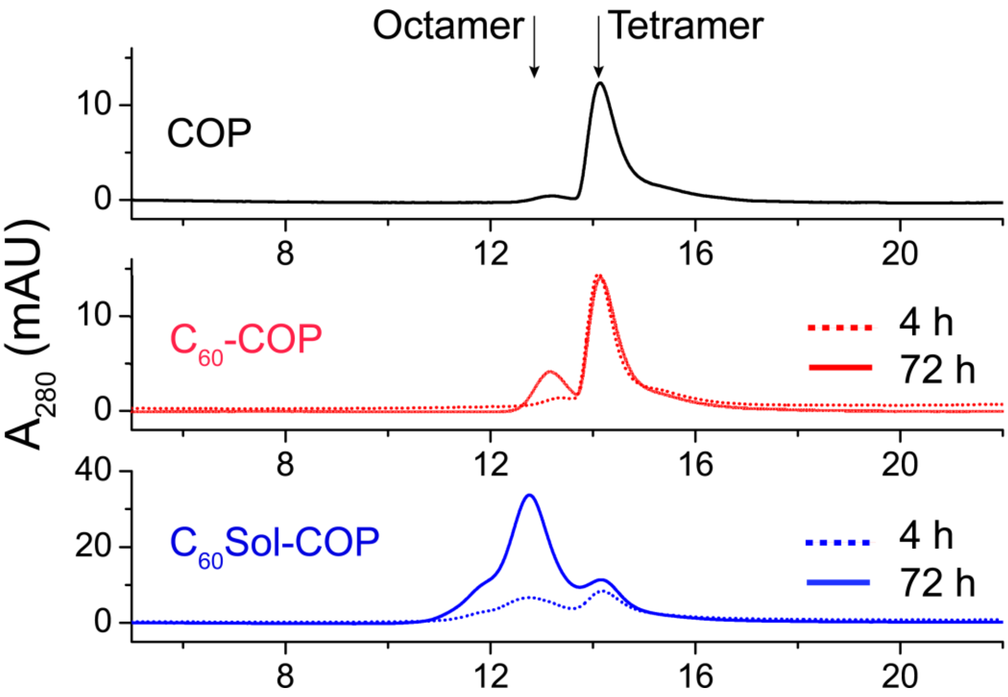

Supplementary Figure 2 | SEC of COP assemblies in association with C_{60} and C_{60}Sol upon different incubation times. C_{60} or C_{60}Sol assemblies were incubated for 4 h or 72 h, and the complex solutions were run through a Superdex 75 10/300 GL gel filtration column. Shown are the resulting UV absorbance profiles at 280 nm versus the elution volume (in mL) of the COP alone (black), C_{60}-COP (red), and the C_{60}Sol-COP (blue). The additional peak that clearly emerges with longer incubation is consistent with an octameric molecular weight (see Supplementary Fig. 9).

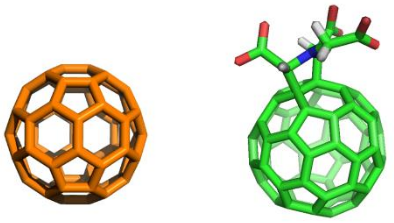

Supplementary Figure 3 | Fullerene ligands used in the study Shown are primitive/bare buckminsterfullerene (orange, left) and a water-soluble fullerene derivative C_{60} pyrrolidine tris-acid or C_{60}Sol (green, right).

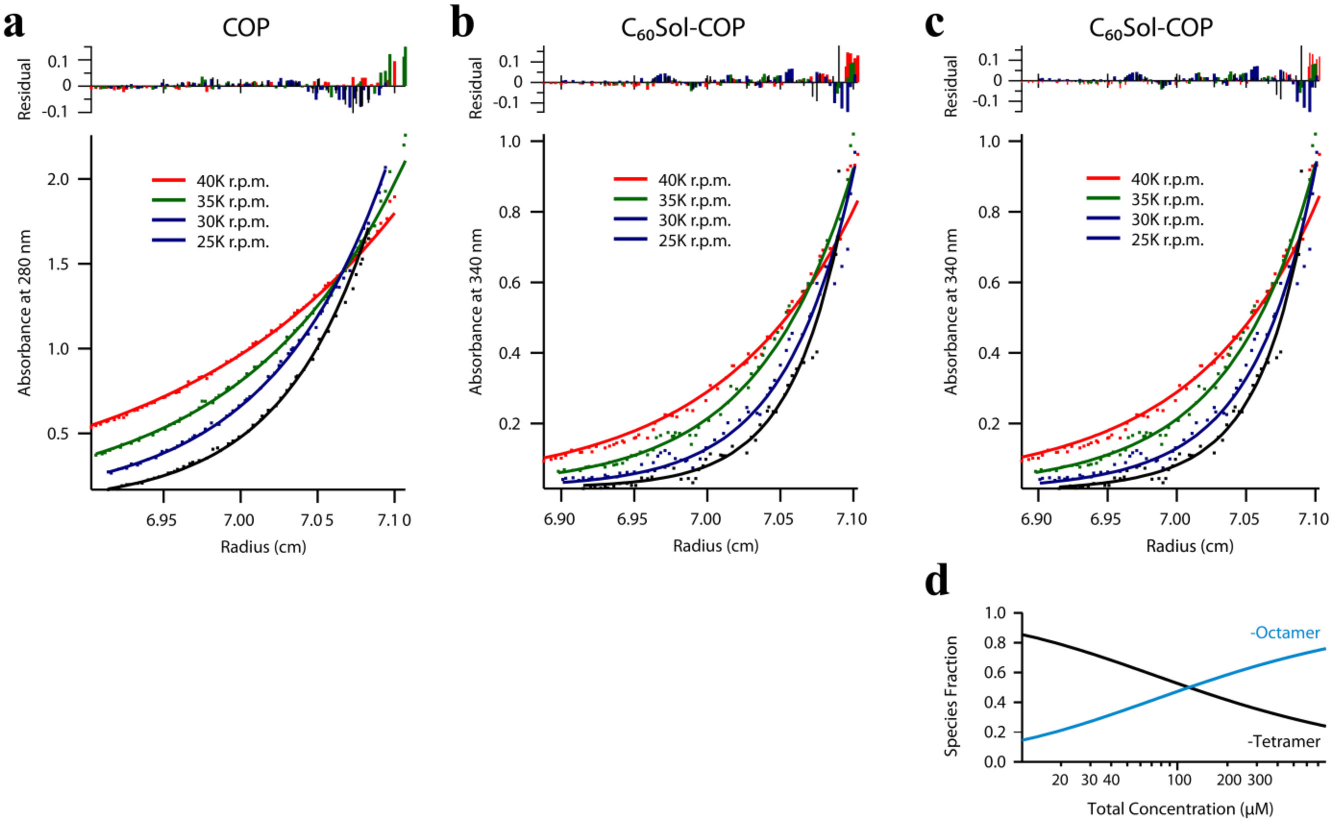

Supplementary Figure 4 | Analytical ultracentrifugation shows that COP co-assemblies into octamers with C_{60}Sol in solution. The COP and C_{60}Sol-COP solutions were centrifuged at four different speeds. Data were globally fit to a function describing sedimentary equilibrium curves. **(a)** Single-species fitting of COP alone yields a molecular weight 13.3 kDa at 280 nm, which is in good agreement with a tetramer. **(b)** Single-species fitting of C_{60}Sol-COP yields a molecular weight 21.9 kDa, which is about 7 times that of a peptide monomer of 3.226 kDa. The disassociation constant between COP tetramer and octamer is 118 μM calculated by a two-species fitting **(c)**. At the applied concentration of COP in solution (about 450 μM as tetramer), the octamer assembly is the predominant species (d). Residuals between calculated and observed data are shown above each fitting plot.

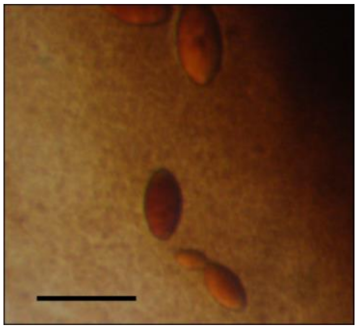

Supplementary Figure 5 | Photograph of C_{60}Sol-COP crystals The scale bar represents 0.1 mm.

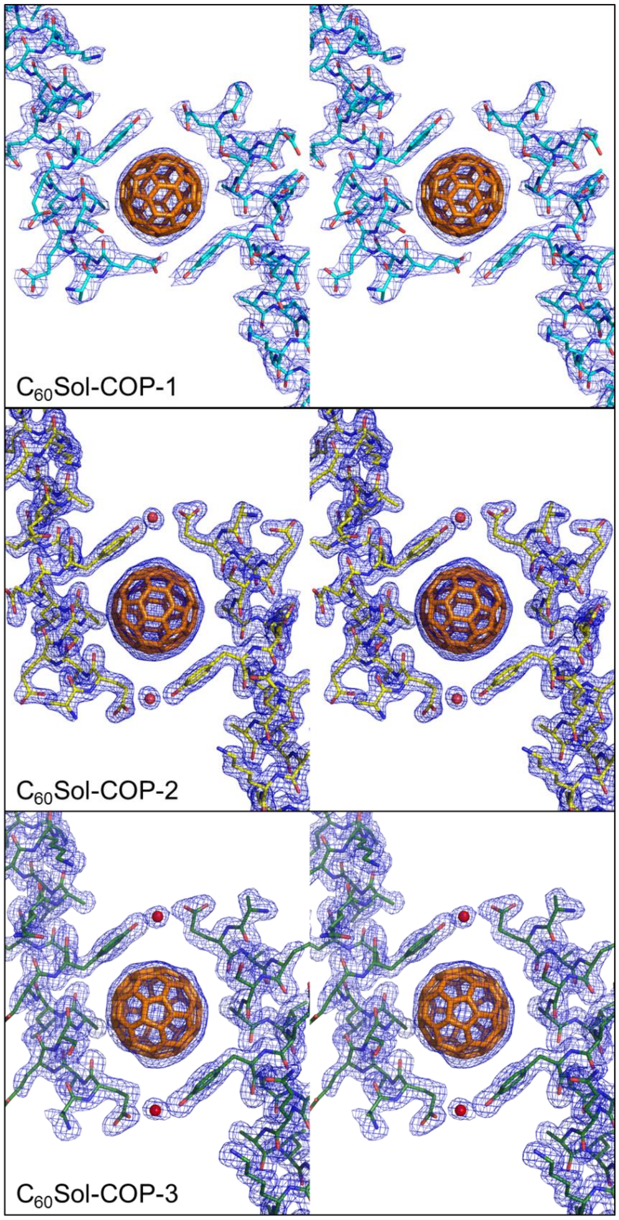

Supplementary Figure 6 | Stereo view of the electron-density map of C_{60}Sol-COP crystals. Stereo images of the region surrounding C_{60} (orange) are shown as $2F_o$-F_c electron-density maps contoured at 1.2σ.

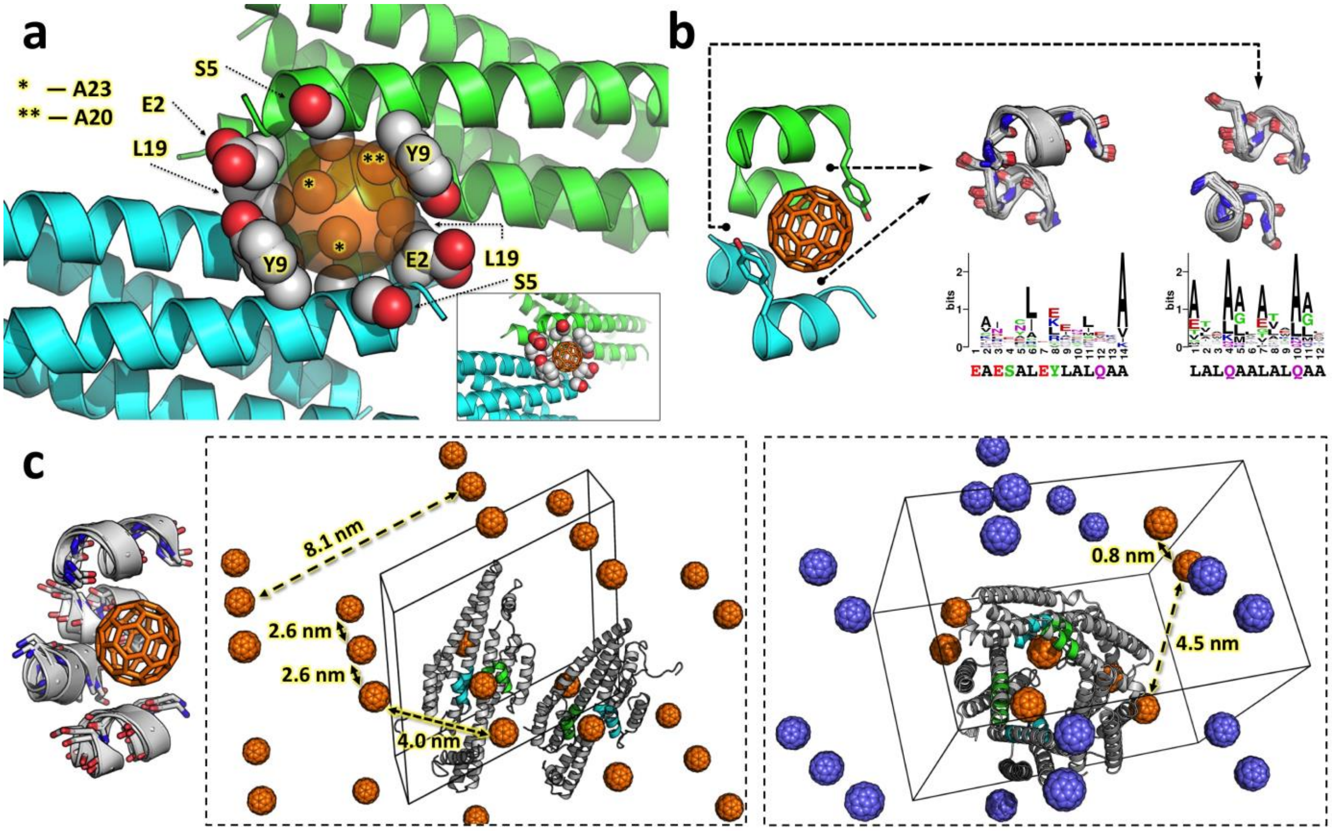

Supplementary Figure 7 | C_{60}-binding mode in the co-crystal represents a general fullerene-organizing motif. a, A close-up of the C_{60}-binding motif. The fullerene is shown in semi-transparent orange surface, with carbon atoms involved in C_{60} packing shown as gray spheres. Key residues are labeled. Cyan and green represent two adjacent COP tetramers in the co-crystal lattice (inset shows a different view angle). The binding region is the only point of contact between individual COP tetramers in the structure. **b,** Analysis of the fragment representing the C_{60}-COP binding motif (left) shows that the protein-protein interfaces needed to build the geometry are highly designable and ubiquitous in nature. Shown superimposed on the right are non-redundant structural examples, from PDB lattices, matching the two unique interfaces (to within 0.3 Å backbone RMSD), along with the apparent sequence preferences extracted from alignments of these matches. Strongly preferred amino-acid choices agree with the sequence of COP from the corresponding region, shown underneath the sequence logos (plots created with WebLogo[1]). **c,** The entire motif, with four comprising segments, also has close analogs in the PDB (left). On the basis of these matches, it is possible to build novel C_{60}/protein lattices (by copying the geometry of the motif in **b**), with two examples shown on the right. In each case, the matching region is shown with the same color scheme as in **b**. Inter-C_{60} distances (shown) vary depending on the parent lattice.

a

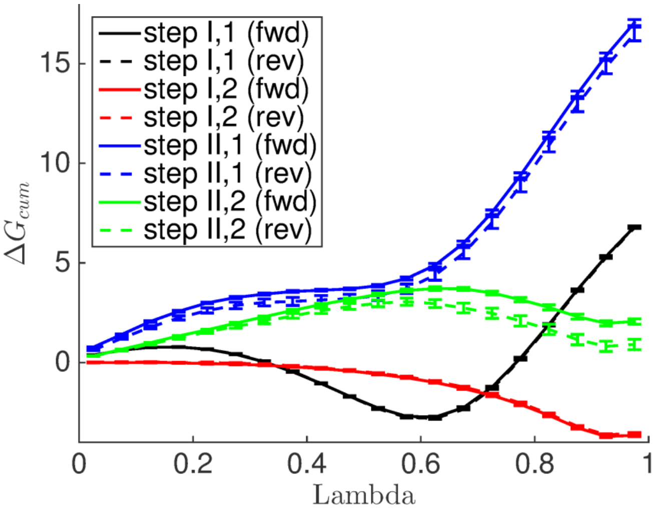

b

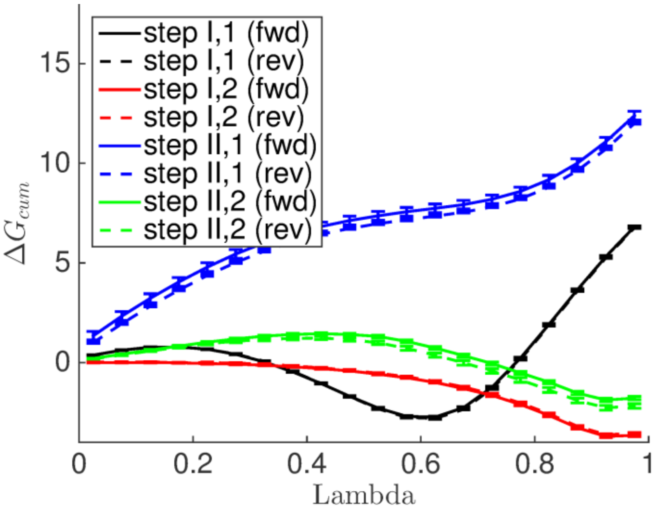

c

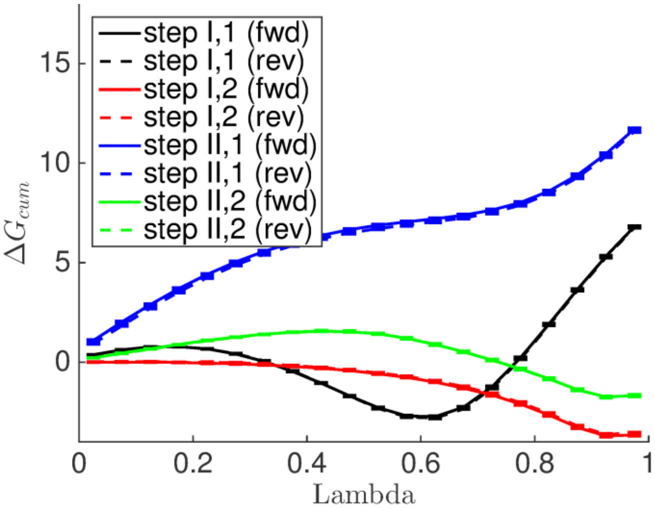

Supplementary Figure 8 | FEP energy profiles for the steps of the modified double-decoupling procedure

Plotted are running free-energy differences as a function of the FEP coupling parameter (0 corresponds to full coupling and 1 to full decoupling). The legend designates each of the four steps by the transformation (I or II) and the step number (1 or 2). Forward and reverse simulations for each step are shown in the same color as solid or dashed lines, respectively. For ease of interpretation, estimates from reverse simulations were negated to correspond to free-energy differences in the decoupling direction. In all cases, final values are well above statistical error (calculated as described in the text), and so is the final estimate of binding free energy. Panels **a**, **b**, and **c** correspond to binding of C_{60} COP, a Tyr residue, or p-methylphenol, respectively.

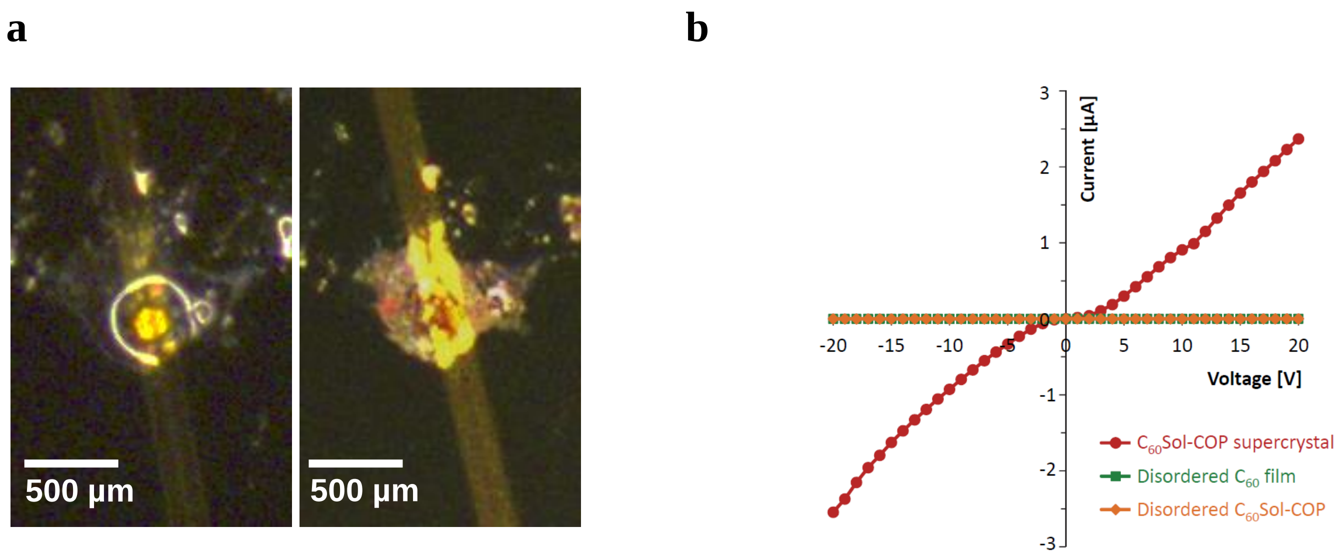

Supplementary Figure 9 | Current-voltage (I-V) characteristic of C_{60}Sol-COP supercrystal.
a, Reflective images show a C_{60}Sol-COP supercrystal on device electrodes before (left) and after (right) destruction under high vacuum. **b,** Current-voltage (I-V) curve of C_{60}Sol-COP supercrystal plotted on a linear scale (red dots). I-V curves of disordered C_{60} film (green squares) and disordered C_{60}Sol-COP (orange diamonds) are also plotted for comparison

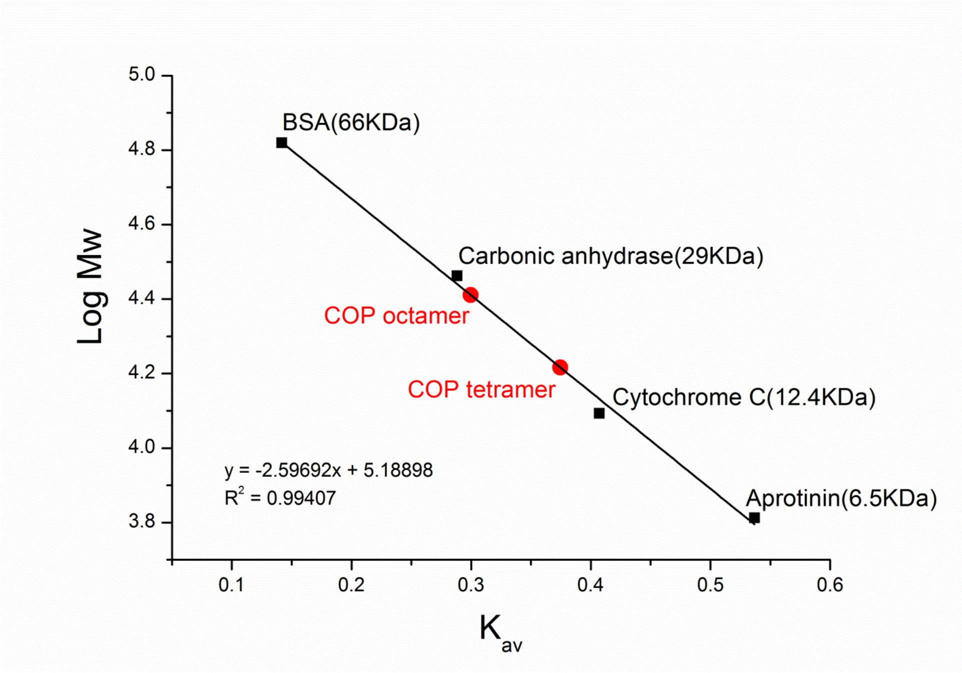

Supplementary Figure 10 | SEC standard curve Plotted are K_{av} values ($K_{av} = (V_R - V_o)/(V_t - V_o)$, where V_R, V_o, and V_t are the retention volume, the interstitial/void volume, and the total volume, respectively) obtained from elution volumes of different protein standards against log molecular weight. Black square boxes correspond to the protein standards used: BSA (M_r 66000), carbonic anhydrase (M_r 29000), cytochrome c (M_r 12400), aprotinin (M_r 6500). Red circles indicate the intersection of K_{av} values of the two peaks in the C_{60}Sol-COP elution profile (Fig. 1c) with the standard curve. This gives estimated molecular weights of ~15,000 Da ~26,000 Da, quite close to 12,742 and 26,204 expected for one COP tetramer and two COP tetramers with one C_{60}, respectively.

Supplementary Tables

Supplementary Table 1. Crick coiled-coil parameters for the *apo* and C_{60}-bound COP structures

Parameter[1]	COP *apo*	COP-C_{60}Sol
superhelical radius, $\boldsymbol{R_o}$, (Å)	6.87	6.87
superhelical frequency, $\boldsymbol{\omega_o}$, (°)	-3.15	-3.37
superhelical frequency, $\boldsymbol{\omega_1}$, (°)	102.4	102.7
pitch angle, $\boldsymbol{\alpha}$ (°)	-14.6	-15.5
helical phase, $\boldsymbol{\varphi_1}$, (°)	-57.9	-59.9
starting heptad position	**b**	**b**
superhelical phase offset for D_2, $\Delta\boldsymbol{\varphi_o}$, (°)	-75.8	-77.9
fitting error, (Å)	0.600	0.395

[1]—see reference [2] for parameter definitions and more information.

Supplementary References

1. Crooks GE, Hon G, Chandonia J-MM, Brenner SE. WebLogo: a sequence logo generator. *Genome research* **14**, 1188-1190 (2004).

2. Grigoryan G, DeGrado WF. Probing Designability via a Generalized Model of Helical Bundle Geometry. *J Mol Biol* **405**, 1079-1100 (2011).
